# Bladder, bowel, and sexual dysfunction are overlooked in the management of neurological conditions: a qualitative interview study

**DOI:** 10.1186/s12883-026-05026-w

**Published:** 2026-06-05

**Authors:** Katie Webb, Gemma Clunie, Graeme Cossou, Holly Roy, Matthew Smith, Marcus Drake

**Affiliations:** 1 Physiotherapy Department, Imperial College Healthcare NHS Trust, London, UK; 2 Department of Surgery and Cancer, Imperial College London, London, UK; 3 University of Plymouth, Plymouth, UK; 4 Neurosurgery Department, University Hospitals Plymouth NHS Trust, Plymouth, UK; 5 Ageing & Movement Research Group, University of Bristol, Bristol, UK; 6 Department of Neurology, North Bristol NHS Trust, Bristol, UK; 7 Department of Urology, Imperial College Healthcare NHS Trust, London, UK

**Keywords:** Bladder dysfunction, Bowel dysfunction, Management, Neurological disease, Sexual dysfunction

## Abstract

**Background:**

One in six people in the UK live with a neurological condition, of which many experience bladder, bowel and sexual dysfunction that are present during the time of neurological diagnosis. However, it not clear whether their bladder, bowel or sexual symptoms are considered when formulating a neurological diagnosis. This study aimed to explore the patients experiences of being diagnosed with and managing neurological conditions and the considerations of their bladder, bowel and sexual symptoms.

**Methods:**

Semi-structured qualitative interviews were conducted with patients with a diverse range of neurological conditions. Interviews were recorded and transcribed verbatim and analysed using reflexive thematic analysis. A patient and public involvement group supported the pilot testing of the topic guide and the data analysis.

**Results:**

Twenty participants (12 female, 1 non-binary and 7 male) aged between 30 and 83 and 13/20 people from the global majority participated in the study. Four themes and ten subthemes were identified: (1) Challenges within healthcare – dismissive encounters with healthcare professionals and systemic pressures directly impacting care. (2) Understanding and Advocacy – insufficient communication, education and expectation management across contexts, “I am the ruler of my own self”- self advocacy is essential and peer support. (3) Emotional and Psychological Impact – Grief – “you go through the grieving cycle; you’ve lost a bit of yourself” and difficulty describing sensations. (4) Sex Matters – Gender differences and sex remains a low priority within healthcare.

**Conclusions:**

The findings demonstrate that during diagnosis of neurological conditions bladder bowel and sexual symptoms are overlooked in building the clinical picture, and the needs of managing these emotionally challenging and difficult symptoms are not being effectively met by the healthcare system at present.

**Supplementary Information:**

The online version contains supplementary material available at 10.1186/s12883-026-05026-w.

## Introduction

One in 6 people in the UK live with a neurological condition [1]. In many neurological conditions, bladder, bowel and sexual function symptoms are present [2], sometimes presenting prior to the neurological diagnosis [3]. Despite this, bladder, bowel and sexual symptoms are often not recognised as neurological in origin and are overlooked when formulating a diagnosis. The limited mention of bladder, bowel or sexual symptoms in the “Suspected Neurological Conditions” NICE guidelines 2019 [4] summarises the lack of recognition that bladder, bowel or sexual dysfunction could be neurological in nature.

Previous research has explored the experiences of people living with neurogenic bladder, bowel and sexual function after having been diagnosed [5–13], in chronic neurological disease such as multiple sclerosis, Parkinson’s disease and spinal cord injury. Despite less common neurological conditions, such as multiple systems atrophy and progressive supranuclear palsy also having known neurological bladder, bowel and sexual dysfunction [2] there is no research to our knowledge exploring their experiences. Moreover, there is a research gap that exists in understanding how the recognition of bladder, bowel and sexual dysfunction as part of the process of reaching a neurological diagnosis varies, let alone appreciating that these are neurogenic and should be treated as such. Subsequently, care for these neurogenic bladder, bowel and sexual dysfunction is often delayed and fails to reflect the needs of the patient [14]. Evidence suggests that individuals presenting with atypical initial symptoms of neurological disease, which may include a predominant picture of bladder, bowel, or sexual dysfunction, are more likely to experience diagnostic delays when compared to those presenting with motor, sensory or cognitive symptoms [15].

Bladder, bowel and sexual domains are included within disease-specific, quality of life questionnaires, such as the non-motor symptom questionnaire for PD [16], the Multiple Sclerosis Quality of Life Questionnaire (MS-QOL) [17] evidencing that there are opportunities for bladder bowel and sexual dysfunction symptom management once diagnosed.

The aim of this study therefore was to explore the experience of being diagnosed with a neurological condition and whether bladder, bowel and sexual symptoms were considered within the initial diagnosis. A secondary aim included the exploration of care and support given to individuals living with neurological conditions to manage their bladder, bowel and sexual dysfunction. The research question is: What are the experiences of being diagnosed and living with neurological conditions and how were bladder, bowel and sexual dysfunction considered when making the diagnosis?

## Methods

### Study design and analysis

This study used qualitative methods to explore the experiences of being diagnosed and living with a neurological condition and its associated bladder, bowel and sexual dysfunction, via semi-structured interviews, using reflexive thematic analysis to identify and interpret patterns of meaning across the data set [18]. The Consolidated Criteria for Reporting Qualitative Research Studies Checklist (COREQ) [19] was used to report the methods and results of this study (supplementary material 1). Braun and Clarke’s values-based reporting guidelines were also consulted to ensure that the results were reporting congruently with qualitative research values [20].

The study received NHS Ethical Approval from the London Fulham Research Ethics Committee (REC 24/LO/0399).

### Participants & setting

Eligible participants had a diagnosed neurological or neurosurgical condition, were over the age of 18 and spoke English. Opportunistic, purposive sampling was used to recruit participants with a diverse range of neurological conditions, impacting various locations in the nervous system from a range of ages, genders and ethnicities (see Table 1). The characteristics of the participants was charted, on a pre-designed framework, throughout the study, to ensure diversity within the study population was met. Participants were predominantly approached in person or via telephone, by the lead researcher (KW) or by members of their clinical team (GC). All participants were recruited from a single, large London teaching hospital in North-West London, in various outpatient and inpatient settings including neurology, neurosurgery, urology and physiotherapy wards and clinics. Participants were identified either directly by the research team, who were not involved in their clinical care, or identified by clinicians within their usual care team, who subsequently informed the research team. The participants identified by their clinical care team were not under the care of the primary researcher conducting the interviews. They were provided reassurance that their identities would remain anonymous throughout reporting and that participation would not impact their clinical care.

### Patient and public involvement (PPI)

Five individuals living with neurological conditions (ages 30–54), including 2 females and 3 males (four global majority) participated in the PPI group influencing the study design, methods of data collection and were integral to the analysis of the data.

### Data collection

Participants consented to take part in the study after being provided with a patient information sheet on the study and were consented by the researcher (KW). Verbal and written consent was confirmed before each interview. Demographic information was provided by each participant by completing a demographic form.

The semi-structured interview topic guide was developed by the research team for this study (Additional file 2) and was piloted with the PPI group, who made impactful amendments to the interview schedule for exploration of symptoms, particularly sexual, to minimise risk of unwelcome intrusiveness.

Interviews were conducted by the lead researcher (KW). These interviews took place either in a private clinical room in the hospital, online via Microsoft teams or via telephone, based on participant preference. Only the participant and the researcher were present during the interviews, except for one participant who attended with a family member for support. The researcher had been involved in the care of one of the participants prior to participation in the research, with explicit reassurance to all participants that their care would not be impacted by their involvement in the study. Audio recordings were transcribed verbatim alongside field notes made during and immediately following interview completion. These documented interactions, feelings and observation from the interviews allowed for further iterations of the topic guides and helped reduce risk of researcher bias. Interviews lasted between 20 and 80 min with a mean time of 40 min. Participants were given the opportunity to contact the researcher after the interview to share any additional information, or repeat their interview, though none chose to do so.

Recruitment to interviews continued until thematic saturation was achieved, defined as the point in which no new themes are recognised during data analysis [18].

### Data analysis

The interviews were pseudonymised prior to being transcribed. All transcripts were checked against the recordings for accuracy by KW. NVIVO QSR International qualitative analysis software (Version 14) was used to store and manage the transcripts and support data analysis (QSR International Pty Ltd, 2018). Data was analysed using Braun and Clarke’s thematic analysis [18]. A single researcher (KW) familiarised herself with the data by reading through transcripts and listening to audio recordings. Analysis began by line-by-line coding of each transcript, identifying sections or passages within the transcripts that might be relevant to the research question. Each transcript was coded twice by the lead researcher. Initial codes were discussed with a second researcher (GC) to compare interpretations of the data [18]. Various rounds of analysis, via in-depth discussion, referring to original data, were used to interpret and establish meaning behind codes, identifying similarities and patterns between participants responses and were clustered together to generate initial themes. An example codes “time taken for bodily functioning tasks” and “adaptive or coping strategies to manage bladder symptoms”, initially were considered as part of the wider impact of the bladder, bowel and sexual dysfunction. However, on further interpretation and consideration of these experiences, they were interpreted as evidence of a loss or grief experiences occurring on a regular basis, including a difficulty in trusting their own bodily functions and impacting on their self-identity. Contrary cases for example female participants who were sexually active were acknowledged and considered by the researchers during analysis.

In a second round of analysis, the codes were clustered together with others that reflected a similar pattern or concept within the data to generate initial themes. Each theme recognised a shared meaning across the datasets, and a thematic map developed to visually illustrate the relationship between themes. The themes, thematic map and nomenclature were analysed with PPI members, senior qualitative researcher (GC) and clinical expert (MD) and revised to arrive at the final iteration. Refinement of the themes and their relationships continued via in-depth discussion and consideration of the meaning behind the theme, referring back to initial codes, until meaningful analysis was achieved [18]. Participants were invited to review the initial thematic framework for commentary to ensure it was representative of their experiences.

### Reflexivity

The lead researcher (KW, research fellow) with a clinical background in pelvic health physiotherapy, did not inform the participants of her clinical role to prevent any biases or assumptions participants may make knowing her profession. Field notes were used to document interactions, guide improvements and mitigate researcher bias [21]. These were reviewed by the study team to address potential bias. As a woman of menstruating age and healthcare professional (KW), the researcher was aware of this influence on the interpretation of the results and actively reflected on this during the analysis with the other researchers to raise self-awareness and self-critique over interpretation of the findings. Additionally, interpretation and analysis of the data were completed by two women of menstruating age (KW & GC), who felt the sexual symptoms and considerations were overt in their presentation. With the lead researcher, with a clinical background in pelvic health, initial interpreting themes from a more organ based approach, the co-researcher (GC), whose clinical speciality outside of this area of work, was able to challenge this silo nature thematic breakdown and challenge the thematic development.

## Results

Thirty potential participants were approached. After twenty interviews saturation was achieved. Ten of the participants were unable to or declined to take part in the research due to ill health or changes in personal circumstances. The population mean age was 52.9 ± 15.3 with 13/20 of the participants being from the global majority and 13/20 being female or non-binary. See Table 1 for full demographic characteristics of the included population.

**Table 1:** Age, sex, gender, ethnicity and disease characteristics of the sample participants

Pseudonym	Sex (Male, female, intersex)	Gender (Man, Woman Non-Binary)	Ethnic Group	Location of condition	Condition
NPOP001	Female	F	White British	Disease affecting peripheral nerve	Pelvic peripheral neuropathy following lower anterior resection and chemotherapy
NPOP002	Female	F	Arab	Disease affecting spinal cord	Cervical and thoracic spinal cord dysfunction caused by nitric oxide induced vitamin B12 deficiency
NPOP003	Female	Woman	Asian- Indian	Disease affecting cauda equina	CES: disc prolapse
NPOP004	Male	Man	Mixed - White and Black Caribbean	Disease affecting cauda equina	CES: lumbar fracture
NPOP005	Female	Woman	Black or Black British – Caribbean	Disease affecting cauda equina	CES: spinal tumour
NPOP006	Female	Woman	Arab Asian	Disease affecting cauda equina	Traumatic sacral SCI
NPOP007	Male	Non-binary	None specified	Disease affecting peripheral nerve	Pelvic peripheral neuropathy following cysto-prostatectomy and hernia repair
NPOP008	Male	Man	White British	Disease affecting peripheral nerve	Pelvic fracture causing dorsal penile nerve injury
NPOP009	Female	Woman	British Pakistani	Disease affecting cauda equina	CES haemangioma
NPOP010	Male	Man	White - British	Disease affecting spinal cord and cauda equina	CES: disc prolapse
NPOP011	Male	Man	White – British	Disease affecting spinal cord	Thoracic spine fracture
NPOP012	Female	Woman	Black or Black British - Caribbean	Disease affecting spinal cord	MS: Cerebral and Thoracic lesions
NPOP013	Male	Man	White - British	Disease affecting spinal cord	Thoracic spine transverse myelitis
NPOP014	Female	Woman	Any Other Ethnic Group	Disease affecting spinal cord	Thoracic spinal dural fistula
NPOP015	Female	Woman	African	Disease affecting brain and spinal cord	Ms: Cerebral, cervical and upper thoracic lesions
NPOP016	Female	Woman	Participant did not wish to disclose	Disease affecting brain	Congenital hydrocephalus
NPOP017	Female	Woman	White - British	Disease affecting brain	MS: Pons lesions
NPOP018	Female	Woman	White - British	Disease affecting brain and spinal cord	MS: Cerebral and thoracic lesions
NPOP019	Male	Man	Black or Black British - Caribbean	Disease affecting spinal cord	HTLV-1
NPOP020	Male	Man	British Kurdish	Disease affecting brain and spinal cord	MS: Cerebral and cervical lesions

*CES* Cauda equina syndrome, *HTLV-1* Human T-lymphotropic Virus 1, *MS* Multiple sclerosis, *SCI* Spinal cord injury

Table 2 illustrates the themes identified from the data, comprising four main themes: *challenges with healthcare*,* understanding and advocacy*,* emotional and psychological impacts and sex matters* each with their associated subthemes. Although the themes were presented separately for clarity, there were evident interconnections that influenced and shaped participants’ overall experiences.

**Table 2:** Interview themes and subthemes

Theme	Subthemes
Challenges within Healthcare	-Dismissive encounters with healthcare professionals -Systemic pressures directly impacting care
Understanding and advocacy	-Insufficient communication, education and expectation management across contexts -“I am the ruler of my own self”- Self-advocacy is essential -Peer Support
Emotional and Psychological Impact	Grief – you go through the grieving cycle, you’ve lost a bit of yourself Alienation from own body Difficulty describing sensations
Sex matters	Gender differences Sex remains low priority within healthcare

### Theme: challenges within healthcare

The below mentioned subthemes relate to the difficulties participants experienced within the healthcare settings including the difficulties navigating the system due to pressures, leaving participants feeling dismissed during healthcare encounters.

### Subtheme: dismissive encounters with healthcare professionals

*“It was making me feel like I was going crazy. I felt like no one believed what I was saying” (NPOP015*,* Female*,* Woman*,* MS)*

During the interviews, multiple participants highlighted having concerns dismissed by their healthcare professionals (HCPs). This occurred at different time points during their condition, but most markedly round diagnosis. Participants felt that if HCPs had listened to them, then their treatment could have commenced sooner and potential progression in symptoms minimised.

*“I feel like if they took my symptoms first seriously*,* then I wouldn’t have ended up exactly how I’ve ended up “ (NPOP003*,* Female*,* Woman*,* CES)*

Similarly challenging experiences identified assumptions related to existing health conditions. This included menopause, diabetes, enlarged prostate and stress, all of which can cause bladder, bowel or sexual dysfunction, being assumed as their cause for the individual, without diagnostic confirmation. This meant that people were receiving inappropriate care and ultimately delayed appropriate management.

*“They didn’t acknowledge it* [bladder symptoms] *whatsoever*,* but if I’m being honest*,* They didn’t acknowledge my bladder “ (NPOP002*,* Female/ Woman*,* cervical and thoracic spine cord dysfunction following nitric oxide induced vitamin B12 deficiency)*

At times, HCPs ignored symptoms that disagreed with their diagnosis. For example, one of the participants reported anal numbness on examination, associated with the sudden onset of bladder symptoms, which were disregarded and led to misdiagnosis of prostate issues instead of transverse myelitis. Moreover, it was highlighted that healthcare professionals failed to recognise bladder, bowel and sexual symptoms to be potentially neurological in nature, which also led to a delay to diagnosis.

*“I think maybe not just always assuming it’s your underlying health issue. I think that’s probably the key” (NPOP012*,* Female*,* Woman*,* MS)*

### Subtheme: systemic pressures directly impacting care

The strain on the wider healthcare systems was evident and directly impacted patient care. Participants described discharge pressures, for example being discharged from hospital without clear understanding of what to expect following diagnosis/surgery. This was also experienced in the delays to follow up and community services, which contributed to individuals feeling the need to be a strong self-advocate and searching for alternative support.

*“The care in the hospital was just unbelievable. It was so good. It’s unreal. But the aftercare has just been non-existent. I’ve had really nothing. I’ve had a few phone calls*,* over seven months” (NPOP011*,* Male Man*,* Thoracic spine fracture)*

*“the NHS is under pressure but after having such a serious injury and having to wait over a year and a half before I start seeing people who specialise in the areas that have affected me*,* It’s disappointing really” (NPOP004*,* Male Man*,* CES)*

These systemic pressures meant that participants recognised the importance of maximising each healthcare encounter. However, discrepancies between healthcare priorities and patient priorities were evident, often leaving participants feeling despondent or disheartened.

*“I know*,* for each doctor*,* time is precious*,* but what’s the use of time if you’re not using it in the right way?” (NPOP016*,* Female/Woman*,* Congenital hydrocephalus)*

*“But they don’t really look much into your file when I go to the GP. I noticed. They don’t look very much in their file then. I don’t know if it is the time constraint*,* you’re sitting there for 15 minutes*,* and that’s it*,* and when I leave*,* there’s something else I should have wanted to say*,* but I didn’t get to say it because the time comes…”.( NPOP019*,* Male/Man HTLV-1)*

### Theme: understanding and advocacy

#### Subtheme: Insufficient communication, education and expectation management, across contexts

It was apparent throughout the interviews that education and information sharing across contexts, including to the patient, their families and between HCPs was sub-optimal. Participants described difficulties carrying out their own research throughout their healthcare journey, starting with a basic lack of understanding of their diagnosis e.g. “cauda equina syndrome”.

“*It was left to my wife and family and stuff to try and find out some information. And it wasn’t very easy to find*,* if you don’t know what you’re looking for.” (NPOP011*,* M*,* Thoracic spine fracture)*.

There was a lack of clarity around prognosis and expectation management for people diagnosed with neurological conditions, particularly for those who underwent surgical procedures, rather than those with progressive disease. Participants felt uninformed of what to expect in terms of recovery from their condition, leaving them feeling anxious and lost.

*“They are coming back now [sensations]*,* but they didn’t tell me how long it’s going to be….They made me believe that it would only be three or four*,* six months*,* sort of thing*,* but it looks like it’s going to be years.“ (NPOP013*,* M Thoracic spine transverse myelitis)*

*“I think just a little bit more information in the hospital. That would be helpful. Because I’m still not aware of how long this can last or what can happen… which is a bit frustrating because you don’t know when you’re going to start feeling better in yourself” (NPOP009*,* F*,* CES)*

People wanted to understand the timelines associated with recovery for their conditions, if relevant, so they could manage their own expectations. One individual presented a positive experience where HCPs gave them sufficient information, and they felt less concerned about their symptoms “*it’s nice to know the details of what to expect”.* It is clearly beneficial for individuals to be well educated on their condition to manage their own expectations and emotions.

Not only was this education and information related to their condition itself but was also around access to support in the community and following discharge from hospital/ in-between appointments. “*You’re kind of left to your own devices*,* you know*,* to just get on with it*”. There was a sense of being isolated once discharged from hospital.

Because of insufficient education and expectation management, many participants explained they had independently researched their condition, mostly online. This resulted in reassurance that they were not alone in their diagnosis but also concern about whether online information was correct. The desire for further support from trusted professionals remained.

*“I ask this ChatGPT and this kind of things a lot of questions to answer me. Maybe it’s not absolutely 100% correct. But in the meantime*,* going to research it to the website and books*,* stories*,* TV.” (NPOP020*,* M*,* MS)*

Some participants also felt there was a lack of meaningful, effective communication and knowledge sharing between primary and secondary care providers. Participants felt at times their primary care providers did not always understand their condition, the potential symptoms, side effects and community-based services available to their patients. Participants described shared responsibility between primary and secondary care for symptom management, but felt that primary care providers were not always aware of the potential symptoms an individual may experience:

*“I do feel I had to justify why I was looking for the Viagra with the GP…when you can see somebody’s had back surgery a month or two before*,* the chances are*,* whenever you’ve had spinal surgery*,* you’re going to be impacting stuff there.” (NPOP010*,* Male/Man*,* CES)*

#### Subtheme: “I am the ruler of my own self”- Self-advocacy is essential

Self-advocacy was central to the experience of most participants, with a direct relationship across sub-themes. Participants emphasised that because of dismissive encounters and lack of information they must be prepared to self-advocate. Many participants reported the need to “fight” to access necessary services and support. to manage and be able to live with their conditions. They described the resilience and strength it takes to advocate for themselves, a process that carries both emotional and physical weight, adding to the burden of an already fatigued individual.

*“I’m not being heard*,* I’m not being listened to*,* I’m not being helped*,* so what’s the point?”* (*NPOP016*,* Female/Woman*,* Congenital hydrocephalus)*

*“I think the key is having to fight but as I said*,* not everyone has the energy to fight or advocate. And I think if you haven’t got the energy to fight or advocate then you’re not really going to get far and that’s not nice.” (NPOP012*,* Female/Woman*,* MS)*.

Participants recognise themselves as the experts in their lived experience which enhanced their ability to self-advocate but may conflict with opinions or priorities of HCPs.

*“I’m the one that understands what is happening for me and what is right and what is wrong” (NPOP016 Female/Woman*,* Congenital hydrocephalus)*

Self-advocacy was particularly pertinent for individuals without mobility issues. These participants reported their sense of a “hidden disability”, explaining that people do not understand or recognise the negative impacts of living with neurological conditions affecting bladder, bowel and sexual function.

*“It feels less*,* because it feels like you might not even being disabled like people who are in a wheelchair. They don’t really understand what we go through like OK if you can walk its done*,* you can manage yourself” (NPOP006*,* Female/Woman*,* SCI)*

#### Subtheme: peer support

A few participants reported benefits from seeking peer support. They described that it is was easier to speak with someone with shared experiences, who could truly empathise in ways that healthcare professionals cannot. These individuals were able to offer non-judgemental counsel, mentorship and advice on how to navigate the challenges and changes that the participants were facing. Peer support reduced feelings of isolation and helped to build connections and a community.

*“It’s easier to talk to someone who’s had the same experiences as you. And obviously*,* you can ask them a lot of things. He even brings up stuff that I wouldn’t even think of*,* which helps” (NPOP011*,* Male/Man*,* Thoracic spine fracture)*

*“Because you’ve got people that are going through something similar to you rather than just talking to a professional. So it’s a different kind of support but more understanding and suggesting” (NPOP012*,* Female/Woman*,* MS)*

### Theme: emotional and psychological impact

The psychological and emotional impact and grief was a universal experience across all aspects of individuals lives.

#### Subtheme: Grief – you go through the grieving cycle, you’ve lost a bit of yourself

“*Sometimes you’re just angry*,* the whole why me. To get out of that cycle can be really hard” (NPOP012*,* Female/Woman*,* MS)*

Grief, although an individualised journey of frustration, anger and acceptance, which differed between individuals and conditions, was a common experience amongst the participants. They experienced grief in relation to change in physical function, be that mobility, or bladder, bowel and sexual function, that influenced social engagement and wider life. For instance, the need to plan outings around access to toilet facilities imposed an additional cognitive and emotional burden, contributing to feelings of low mood among participants. Additionally, bladder, bowel and sexual dysfunction impacts on people’s career choices, progression and access to work, engaging with hobbies, socialising and starting relationships, greatly affecting individual’s identities. Grief, in varying degrees, was evident across all individuals, independent of condition duration, indicating a continuous experiential process.

*“.this has been life changing for me and sometimes you know I have a good old cry because I want my old life back and I realise I’m not really going to get my old life back. It’s not going to happen. So*,* I’m just trying to make the best of what I’ve got.”* (*NPOP004*,* Male/Man*,* CES)*

*“you kind of go through the grieving cycle of you’ve lost a bit of yourself and the same as if you lose someone*,* and they’ve passed away you go talking*,* if you have a health issue you are grieving that bit of yourself that’s gone. Particularly if it’s a long-term health condition that bit of you is gone*,* you then have to change and adapt and accept. And that’s hard*,* that’s very hard.” (NPOP012*,* Female/Woman*,* MS)*

Grief was also frequently associated with changes related to sexual activity. Individuals of all sex and gender.

expressed grief regarding alterations in sexual experience, including changes in sensation that affected sexual involvement, the loss of spontaneity due to required adaptations and a shift in the role of sex within their relationship, for both them and their partners.

*“I used to enjoy it* [sex] *a lot*,* and then my diagnosis happened. I think part of it is to do with grief*,* my own*,* and then my husband’s grief*,* so that also colours it.*” (*NPOP015*,* Female/Woman*,* MS)*

#### Subtheme: alienation from own body

Finally, there was an overwhelming sense of alienation in the form of lack of trust or control in one’s own body that was jointly experienced between the interview participants. Participants described a distance from their own body because of bladder and bowel symptoms, referencing a lack of understanding and ability to control their own body. This results in an inability to trust or rely on their bodily functions. Change in organ sensation in people living with neurological or neurosurgical conditions contributed to this sense of alienation.

“it feels, it feels alien.” (*NPOP004*,* Male/Man*,* CES)*

“You’re like somebody who’s watching somebody’s life, as opposed to actually somebody who’s feeling” (*NPOP018*,* Female/Woman*,* MS)*

#### Subtheme: difficulty in describing sensations

An interesting theme was the difficulty that people have in describing changes to their bodily sensations and the great variability in descriptors and vocabulary used by participants. Often, analogies or comparators were used, for example “like Christmas lights”, “at the dentist and you touch your face”, which require greater interpretation and clarification with healthcare professionals to interpret. This highlights potential challenges around developing standardised assessments using medical terminology, when peoples’ experiences are so subjective and individual, as has been previous recognised [22].

### Sex matters

Sex matters to patients and noticeably intersects with the other themes recognised within the interview data specifically related to dismissal, insufficient information on how to manage sexual function changes and grieving the differences in sexual experiences.

#### Sub-theme: Gender differences

Throughout the interviews, there was a clear difference in individual attitudes and behaviours as well as healthcare support for sex in women compared to men and non-binary participants.

Firstly, men and non-binary participants were aware of, or sought support with adjuncts or adaptations to engage with sex. This included going through time-consuming routines to be able achieve an erection or find pleasure in sex.

“*This may sound odd*,* but I’ve got a routine*,* how to get an erection now. I’ve worked it out.” (*NPOP004, Male/Man *CES)*

*“Some of my favourite areas no longer are there in a sense*,* they can’t be stimulated to heighten the sexual experience*,* so the whole of the sexual act*,* I’ve had to rethink…” (NPOP007*,* Male/Non-Binary*,* Peripheral neuropathy)*

In comparison to the women, men and non-binary participants had far greater self-efficacy and placed greater importance on sex with many of them having actively attempted to re-engage with sex.

However, many of the women described being unmotivated to engage in sex, expressing fear of causing harm or uncertainty about how to make sex pleasurable again, which led some to feel that it was no longer “worth it”. This demonstrated a clear contrast between genders in their understanding and awareness of the options, adaptations, and strategies available to maintain sexual enjoyment, pleasure, and a sense of worthwhileness.

*“I gave up*,* well I didn’t give up sex but I stopped*,* good*,* more than 10 years ago” (NPOP001*,* Female/Woman*,* Peripheral Neuropathy)*

*“reaching an orgasm is difficult*,* so it feels like the payoff for the act is just not worth it.” (NPOP015*,* Female/Woman*,* MS)*

“*how do I feel about sex? I have no sex drive*,* none. Poor husband. I’d rather pull my eyelashes out. I don’t know*,* I’ve got no desires. None. Which is really sad. Which is really*,* really sad” (NPOP018*,* Female/Woman*,* MS)*

Women described sex differently depending on their life stage. For those of menstruating age, who were still sexually active, most felt sex had “become a chore…I don’t like it, it’s just a box that you tick.” For post-menopausal women, most referred to sex as something you might want rather than need and seen as shameful to engage with, on the contrary to the feelings of most male participants.

*“I did find a few old vibrators. I thought I’d just have to go*,* see if they work*,* and they did*,* but I soon stopped that*,* because they would just empty my bladder instantly… I haven’t done that because I thought*,* that’s disgusting. Little old woman with a vibrator*,* it’s disgusting.” (NPOP014*,* Female/Woman*,* Spinal dural fistula)*

It was highlighted that perhaps “couples counselling and sexual therapy” would be beneficial “so it [sex] will become less like a function and more like something we want to do”.

Several women de-prioritised sex, perceiving it as another symptom or problem to manage, one that might lead to further appointments and additional burden. This highlights a gendered difference in prioritisation, with women viewing sex as an extra demand on top of managing multiple symptoms and healthcare demands.

#### Sub-theme: sex remains a low priority in healthcare

In addition to personal differences, there were clear differences in the prioritisation of sex by healthcare professionals, with women disproportionately deprioritised compared to men and non-binary participants. Women explained that their HCPs had not asked about sex and felt they needed to raise the subject of sex themselves. However, the gender of the HCPs influenced whether participants, particularly women, felt comfortable to ask questions about sex.

*“The doctors that I’ve seen*,* the various neurologists*,* it’s never really something that is ever discussed*,* and sometimes*,* depending on your neurologist*,* if it’s a male neurologist*,* then you don’t really want to discuss it” (NPOP015*,* Female/Woman*,* MS)*

*“I’m comfortable talking about it with certain people. I’m not so comfortable talking about it with other people” (NPOP016*,* Female/Woman*,* Congenital hydrocephalus)*

Interestingly despite sex remaining a taboo subject within healthcare consultations, most participants spoke with ease about sex during the interviews and were keen to engage in the topic discussion.

## Discussion

This study aimed to explore the experiences of being diagnosed and living with a neurological condition and its associated bladder, bowel and sexual dysfunction particularly related to the consideration of these symptoms whilst formulating their diagnosis. Despite many presenting with bladder, bowel and sexual symptoms around the onset of their conditions, at times, these symptoms were overlooked or dismissed. Some participants also identified how they felt that earlier recognition of these symptoms may have led to prompter management and the perception of possibly better outcomes.

A key theme reported by participants is the requirement to self-advocate to receive the diagnosis, management and care they require. This is in line with the findings of a nationwide survey of people living with neurological conditions. Whilst 1 in 3 felt satisfied with their care needs, 48% did not feel their healthcare system supported them [23]. A study exploring support for pelvic floor dysfunction in multiple sclerosis reported that participants required “relentless self-advocacy” to receive adequate care. This reinforces the profound physical and emotional need for patients with neurological bladder, bowel and sexual symptoms to explain and validate their experiences [14]. In stroke patients, another group of neurological patients who experience bladder, bowel and sexual dysfunction, multiple factors have been identified as influencing self-advocacy. These include, but are not limited to, personal factors such as a diminished sense of autonomy, disease severity, and a heightened self-perceived burden [24]. Given the substantial effort and emotional energy required for continuous self-advocacy, concerns have been raised regarding potential inequities in care for individuals who are unable to advocate effectively for themselves.

Participants in these interviews identified peer support as a valuable resource, which supports existing evidence that peer support provides possibilities for counselling, fostering a sense of community, improving quality of life and offering advocacy-based support [25]. The under-utilisation of peer support in healthcare represents missed opportunities both for individuals living with a condition, who may benefit from non-judgemental support, and for healthcare providers, who could optimise care by integrating non-clinical resources, without adding burden to the healthcare system. Greater collaboration across sectors has the potential to benefit all stakeholders.

Grief was a consistently exhibited theme across all interviews, irrespective of the duration of each participant’s condition, related to bladder, bowel and sexual symptoms impacting on quality of life. Biological disruption associated with a neurological condition may lead to alienation from one’s own body, shaping an individual’s self-perception and contributing to the construction of an illness-related identity. Both bodily alienation and identity-related changes can be conceptualised as embodiments of grief. The findings highlight the need for enhanced psychological and mental health support as a key component in the management of neurological conditions, acknowledging the enduring nature of grief in this population. Mental health support has been set as a priority for future service provisions by the National Neurosciences Advisory Group [26].

As identified, people with neurological conditions experience bladder, bowel and sexual symptoms [2, 3], which at times can be ignored or minimised. The dismissal of these symptoms during diagnosis has been further highlighted in the interviews. Therefore, greater awareness is required to prevent the delay to recognising bladder, bowel and sexual symptoms as neurological in nature and further delaying diagnosis and treatment. The NHS England Getting It Right First Time campaign for neurological conditions has set out a roadmap to improve specialist access to inpatient and outpatient neurological care with the intention of “getting it right first time” and reducing avoidable healthcare errors [27]. The reasons for dismissal amongst healthcare professionals are likely complex. One issue may be a lack of time to cover pelvic symptoms, with the recommended follow up for a neurology patient in the UK being 15–20 min, which may be filled with other pressing discussion topics, for example the use of disease modifying therapies in MS. Another issue may be the lack of familiarity in assessing (in particular differentiating from non-neurogenic co-morbidity) bladder, bowel and sexual dysfunction and minimal confidence with managing these symptoms. Neurological examination of pelvic structures can increase the diagnostic yield during diagnosis, yet this is covered minimally in UK neurological and neurosurgical training, with the competence of examination more firmly focused on limb and cranial nerve structures.

The interviews revealed that many women were not motivated to address or did not feel their sexual function was worth exploring, because of the changes associated with their conditions and it is well known that fear of urinary or faecal incontinence impacts sexual function and pleasure [28, 29]. Sexual function is inherently embodied and constitutes part of one’s identity. Given the changes in physical embodiment, bodily trust, and self-identity experienced by people with neurological conditions, these changes are likely to affect intimacy, sexual engagement, and relationship dynamics. Although the themes “grief” and “sex matters” were closely related, the authors felt that combining them would risk oversimplifying participants’ broader experience of grief. The authors positionality helped reveal the relationship and differences between the themes that were developed.

However, if healthcare professionals were more proactive and skilled in supporting female patients, comparable to the approach often taken with male patients, women might better recognise that they also have the right to experience sexual pleasure and enjoyment. However, it is well-known that healthcare professionals do not prioritise sexual function and it is often the responsibility of the patient to introduce sex as a topic during consultations [30, 31]. Despite most of the female participants having not discussed their sexual function with their healthcare professionals, many were happy to discuss this during their interviews. This is important to consider, as it is likely that a number of factors contribute to this gap, including fear of causing offense, time pressures and a lack of knowledge of how to provide any subsequent advice. Consequently, the lack of discussion about sexual health in clinical practice constitutes a missed opportunity to meet the sexual function needs of individuals with neurological conditions [30], and highlights a gap in patient-centred care, especially considering the lifelong and potentially progressive nature of these disorders. It is worth noting that one participant (NPOP013, Man, Male) had an in-dwelling catheter in-situ, which impaired his ability to engage in sexual relations and two other participants (NPOP006 and NPOP009) reported using intermittent self-cathetersation (ISC). Neither directly referred to ISC impacting their sexual lives.

Generally, there appeared to be greater knowledge and uptake of available adjuncts to support with male sexual function when compared to female sexual function. The imbalance in adjunctive support and awareness is exemplified in literature discussing the management of sexual dysfunction for individuals living with neurological conditions. Greater options for management of male sexual dysfunction are discussed compared to the dearth of support for women living with neurological sexual dysfunction [32]. This reflects the ongoing sex and gender difference in the awareness, support and management of female sexual dysfunction, within both research and clinical practice. In addition to healthcare adjustments, greater research and increased awareness, education and interventions among women and healthcare professionals are required to optimise the use of adjuncts and improve the management of female sexual dysfunction.

Priorities outlined by the national neurosciences advisory group, a multiprofessional and patient collaboration, report direct care should be personalised and individualised and so sexual function should be included as part of holistic patient-centred care. Patient Concern Inventories, previously developed and implemented in head and neck cancer care [26], represent a potential model for adaptation in neurological services. Such tools could enable patients to set their own appointment agendas, highlight their priorities, and facilitate recognition of symptoms associated with their neurological condition.

The study population represents the diversity of people living in North-West London, which is one of the most ethnically diverse regions in the UK [33]. We acknowledge that people’s experiences could be affected by cultural factors, that may not be fully captured within the study.

### Limitations

This study has several limitations. Firstly, participants were recruited from a single hospital, and included a relatively small population, which may have led to selection bias in the recruitment process. Due to the heterogeneity of the population, particularly related to neurological condition, generalisability may be limited. The majority of participants had spinal pathologies, and the impact in cerebral or neurodegenerative disorders is important to consider. Similarly, the study population did not include individuals with Parkinson’s Disease, where hypersexuality may be experienced. This situation would vary from our current results. However, the similarity in experiences across conditions and care pathways demonstrates a common and shared experience despite the patient heterogeneity. Although the included participants were diverse in terms of gender and ethnicity, the influence of cultural and religious beliefs around bladder, bowel and sexual symptoms were not explored during the interviews, which is likely to influence the findings.

## Conclusion

To our knowledge, this is the first qualitative study to explore the consideration of bladder, bowel and sexual symptoms in relation to diagnosis of neurological conditions. Bladder, bowel and sexual dysfunction are often dismissed or assumed to be related to other, pre-existing conditions, which led to delay in diagnosis and subsequent management of neurological conditions. Those living with neurological conditions must advocate for themselves strongly to receive care they need because these symptoms are not recognised as being related to their neurological conditions. Healthcare professionals ought to be providing non-judgemental counsel and support for individuals living with and managing neurogenic bladder, bowel and sexual dysfunction. There remains a need to improve the support for female sexual health and wellbeing.

## Supplementary Information

Supplementary Material 1.

Supplementary Material 2.

## Data Availability

The data that support the findings of this study are available on request from the corresponding author. The data are not publicly available due to privacy or ethical restrictions.

## Abbreviations

CES: Cauda equina syndrome
COREQ: Consolidated Criteria for Reporting Qualitative Research Studies Checklist
F: Female
HCPs: Healthcare Professionals
HRA: Health Research Authority
HTLV-1: Human T-lymphotropic Virus 1
M: Male
MS: Multiple sclerosis
NICE: National Institute for Health and Care Excellence
PPI: Patient and Public Involvement
SCI: Spinal cord injury
