# Supplementary material for: Bladder, bowel, and sexual dysfunction are overlooked in the management of neurological conditions: a qualitative interview study
Source: BMC Neurol. 2026 Jun 5;26:503. doi: 10.1186/s12883-026-05026-w (PMC13455156; doi:10.1186/s12883-026-05026-w)
Supplement: Supplementary file 1 [file 12883_2026_5026_MOESM1_ESM.pdf]

Interview Topic Guide

Introduction

[Before turning on recorder]

- Introduce myself
- Review project and interview process again and confirm participants are happy to take part
- Reiterate no right or wrong answer, can stop at or take a break at any time and do not need to answer any questions they don't feel comfortable to
- Reminder that I will be audio recording and might write things down to come back to
- Interview will take roughly an hour
- Are they in a confidential space to speak to discuss these topics, if online or telephone interview, and is today still a good day to complete interview with their condition?
- Any questions before we start?

Please note that this guide only represents the main themes to be discussed with the participants as and as such does not include all the various prompts that may also be used (some examples given for each question). Non-leading and general prompts will also be used such as "can you tell me a little bit more about that?" and "What does that look like for you?"

Establishing Rapport

1. Please could you tell me a little bit about your [neurological] condition and your experience was like of being diagnosed?

Prompts:

What led you to seek medical attention?

Could you tell me a little bit more about what was discussed?

How do you feel the experience went?

What went well?

What could have been improved?

How did you feel about the diagnosis?

2. **Bladder symptoms**

I am now going to ask you some questions with regards to your bladder symptoms.

Can you describe the bladder or urinary problems you experience with your condition and any other feelings or differences you experience alongside bladder/urinary symptoms?

Prompts:

Prompts: What is it like when you want to pass urine? How do you pass urine? What are the changes you have noticed when you want to pass urine?

Prompts: What is it like when you want to control your urine? How are you aware when you have had an accident? What are the changes you have noticed when you want to control urine?

Prompts: How do you become aware you need to pass urine? What are the changes you have noticed with the feeling to your bladder?

3. **Bowel symptoms**

The next section will be asking questions around your bowel symptoms.

Please can you describe the bowel or anal problems you experience with your condition and any other feelings or changes you experience alongside bowel/anal symptoms?

Prompt: Can you explain any changes in your ability to control stool/poo or wind?

Prompt: Can you explain any changes in your ability to open your bowels/go for a poo?

Prompt: Are you aware when you need to open your bowels? How are you aware of this? Are there any changes you have noticed with the feeling around you bottom or anus? What are they?

4. **Genital symptoms**

The next questions are about changes to your genitals, including during sex. If you are uncomfortable with answering any question, we can move on to the next one. Do you want to continue or skip to the next section?

Please can you describe any changes you've experienced to your genitals with your condition?

These can be both during sexual activity and/or outside of sexual activity?

Prompts: What are the changes you have noticed to the sensation to your genitals? When do you experience these sensation changes? What are the changes you have noticed?

Prompts: Have you noticed a change in your ability to achieve climax or any other differences during sexual activity? Please can you explain these?

Prompts:

Could you tell me about any changes you experience outside of sex?

Could you tell me about any changes during sex?

Please could you tell me about your ability to achieve orgasm and or ejaculate?

Please could you tell me how this has impacted your sexual activity?

5. **Other pelvic floor/ pelvic symptoms**

Are there any other symptoms related to your pelvic organs or pelvic floor that you feel we should know about?

Prompt: Any other change in relation to you bladder, bowel. genital sexual or pelvic function that you have not had the opportunity to share?

6. **Considering all we have discussed, please could you explain how your bladder, bowel and/or sexual symptoms were considered as doctors were working out your neurological diagnosis?**

Probes:

How were these symptoms considered during the diagnosis?

What did you think went well?

What do you think could have improved?

Closing

7. **Is there anything you think we have not discussed in relation to your bladder, bowels or sexual and genital function and the relationship with your diagnosis that you'd like to share?**

We have had a great discussion today, you have given me great insight into your experiences, which will all remain anonymous. *Thank you very much for your time and the information you shared today, I appreciate that it is a sensitive topic to discuss.*

When we reviewed this interview plan with people with neurological conditions, they felt it might be helpful to offer our email address, in case you had any additional thoughts after the interview. Would you like our email address?

Finally, once we have published our research findings we can contact you with the published paper and results for your interest. If this is something you are interested in.
